# Mobile health solutions: An opportunity for rehabilitation in low- and middle income countries?

**DOI:** 10.3389/fpubh.2022.1072322

**Published:** 2023-01-24

**Authors:** Bruno Bonnechère, Oyene Kossi, Jean Mapinduzi, Jules Panda, Aki Rintala, Susanne Guidetti, Annemie Spooren, Peter Feys

**Affiliations:** ^1^REVAL Rehabilitation Research Center, Faculty of Rehabilitation Sciences, Hasselt University (UHasselt), Hasselt, Belgium; ^2^Technology-Supported and Data-Driven Rehabilitation, Data Science Institute, UHasselt, Hasselt, Belgium; ^3^ENATSE, National School of Public Health and Epidemiology, University of Parakou, Parakou, Benin; ^4^INSP, Institut National de la Santé Publique, Bujumbura, Burundi; ^5^CKAO-AMAHORO, Cabinet de Kinésithérapie et d'Appareillage Orthopédique, Bujumbura, Burundi; ^6^University of Lubumbashi, Lubumbashi, Democratic Republic of Congo; ^7^Institut de Recherche en Science de la Santé, Lubumbashi, Democratic Republic of Congo; ^8^Faculty of Social Services and Health Care, LAB University of Applied Sciences, Lahti, Finland; ^9^Department of Neurobiology, Care Sciences and Society, Division for Occupational Therapy, Karolinska Institutet, Stockholm, Sweden; ^10^Women's Health and Allied Health Professionals Theme, Medical Unit Occupational Therapy and Physiotherapy, Karolinska University Hospital, Stockholm, Sweden

**Keywords:** mHealth, rehabilitation, care, telemedicine, public health

## Abstract

Mobile health (mHealth) development has advanced rapidly, indicating promise as an effective patient intervention. mHealth has many potential benefits that could help the treatment of patients, and the development of rehabilitation in low- and middle-income countries (LMICs). mHealth is a low-cost option that does not need rapid access to healthcare clinics or employees. It increases the feasibility and rationality of clinical treatment expectations in comparison to the conventional clinical model of management by promoting patient adherence to the treatment plan. mHealth can also serve as a basis for formulating treatment plans and partially compensate for the shortcomings of the traditional model. In addition, mHealth can help achieve universal rehabilitation service coverage by overcoming geographical barriers, thereby increasing the number of ways patients can benefit from the rehabilitation service, and by providing rehabilitation to individuals in remote areas and communities with insufficient healthcare services. However, despite these positive potential aspects, there is currently only a very limited number of studies performed in LMICs using mHealth. In this study, we first reviewed the current evidence supporting the use of mHealth in rehabilitation to identify the countries where studies have been carried out. Then, we identify the current limitations of the implementation of such mHealth solutions and propose a 10-point action plan, focusing on the macro (e.g., policymakers), meso (e.g., technology and healthcare institutions), and micro (e.g., patients and relatives) levels to ease the use, validation, and implementation in LMICs and thus participate in the development and recognition of public health and rehabilitation in these countries.

## Introduction

According to the World Health Organization (WHO), “health is a state of complete physical, mental and social wellbeing and not merely the absence of disease or infirmity” (1). The enjoyment of the highest attainable standard of health is one of the fundamental rights of every human being without distinction of race, religion, political belief, economic, or social condition (2). If health is a human right and human rights are “rights held by individuals simply because they are part of the human species” then—according to Ooms et al.—“all people, regardless of where they live, should be entitled to the same collective efforts that can protect or improve their health” (3). Health services encompass all services concerned with promoting, maintaining, and restoring health. These services encompass both individual and population-based healthcare (4).

The WHO emphasizes the need of implementing methods for health promotion, prevention, and rehabilitation, as well as strengthening health information systems, evidence, and research (5). The accessibility to high-quality healthcare, including rehabilitation facilities, has thus been defined as one of the pillars of the sustainable development goals (SDGs goal 3) (6). Rehabilitation strategies consisted of a collection of interventions designed to maximize functioning and reduce disability in individuals with health conditions interacting with their environment. Rehabilitation should be extremely person-centered, which means that the interventions and approaches chosen for each individual should be based on their goals and preferences (7).

Rehabilitation professionals include physiotherapists, occupational therapists, speech and language therapists and audiologists, orthotists and prosthetists, clinical psychologists, physical medicine and rehabilitation physicians, and rehabilitation nurses, among others, and may be administered in different settings, including inpatient or outpatient hospital settings, private clinics, and community settings such as the patient's home (8). In addition to their central role in rehabilitation, it has been shown that physiotherapists are well-positioned within the healthcare system to help minimize chronic illness by frequently screening and managing risk factors for chronic diseases (healthy living medicine) (9). However, countries with the lowest relative need have the most health personnel, whereas countries with the highest health burden must make do with fewer staff (10) and often lower educational level of healthcare professionals (11).

While the bulk of these limited human and financial resources is historically directed toward infectious illnesses, the management of chronic diseases receives just a tiny portion. For example, 80% of stroke cases are reported in low- and middle-income countries (12) [(LMICs)—note that thresholds, definitions, and list of the countries classified according to gross national income are presented in Supplementary Table 1] (13). This is regretful as the WHO is currently highlighting functioning as an important dimension of health besides mortality and morbidity, and nowadays considers rehabilitation as the health strategy of the twenty-first century (14), and having a healthy population is a key to sustainable development. Note that the International Classification of Functioning (ICF) was launched much earlier than the current pledge for rehabilitation for all (5), and the minority perspective for people with disability was targeted (15). According to the WHO, one of the most significant constraints on the rehabilitation process is a lack of access to specialist facilities or healthcare personnel (8).

The use of mobile technology and eHealth may provide an alternative to the aforementioned limitations of care or serve as a complement to existing rehabilitation programs. At least in high-income countries, the development and implementation of mHealth enhance the scope and potential of the healthcare sector (16–19). Mobile technology and eHealth have been of great importance in the context of the COVID-19 pandemic during the different lockdowns and when physical restrictions were imposed and access to rehabilitation services and care was limited (20).

mHealth is a low-cost option that does not need rapid access to healthcare clinics or employees. This might have a huge potential in LMICs to overcome the lack of healthcare professionals, advance rehabilitation, and minimize health inequalities by improving access to rehabilitation, either remotely (due to a scarcity of facilities particularly in rural regions) or financially (21). These aspects are particularly crucial for neurological populations, as these patients need long-term rehabilitation and their disabilities lead to transport or financial issues. mHealth solutions not only can be utilized by using smartphones or tablets but also can be combined with (low-cost) wearable sensors. Mobile health technologies is an umbrella term that has been defined as the use of “wearable, portable, or domestic-integrated devices that can provide objective measures and that include digital applications, as well as body-worn (adhered to a body surface, mainly inertial measurement units) or frequently used patient-centered devices (e.g., smartphone and keyboard)” (22). The use of mobile health technology can, of course, widen the scope of potential applications but it also comes with a downside such as higher cost, more complex to develop integrated solutions, and potentially more troubleshooting to deal with as different technologies are being used. Therefore, in the next part of this study, we will focus on mHealth technology used on smartphones and tablets only.

## Current situation

To evaluate the current use of mHealth solutions across the world, we performed a second analysis of the latest reviews summarizing the efficacy of mHealth applications for stroke (16), healthy aging (17), Parkinson's disease (18), and multiple sclerosis (19). A total of 132 studies were included in this analysis.

### Level of evidence

For stroke, the apps have widely varied content to meet the needs of persons with stroke; however, the studies are generally preliminary in nature, focusing on development, usability, and initial pilot testing (16). Different mHealth applications were identified with heterogeneous content including gamification, monitoring of physical activity, and physical exercises including mobility and motor functions. Positive effects or trends were observed for upper and lower extremity functioning, physical activity, and activities of daily living (23).

For the geriatric population, out of 40 studies, 15 studies (38%) found mHealth to be at least as effective as non-mHealth interventions (56% of the 27 studies with a control group), 11 studies (41%) found mHealth interventions were more effective than non-mHealth interventions, and one study (4%) reported beneficial outcomes in favor of the non-mHealth interventions. Simple interventions are more likely to be feasible for older patients receiving geriatric rehabilitation, especially, in combination with other non-mHealth interventions (17).

For Parkinson's disease, a meta-analysis was performed (18). The results of this meta-analysis show that with respect to PD severity, compared with usual care, mHealth intervention was beneficial in lowering motor impairment of patients with PD significantly [mean difference (MD) = −2.27, 95% confidence interval (95% CI) −4.25 to −0.29, *p* = 0.02], rather than mental status (MD = −0.98, 95% CI −2.61 to 0.65, *p* = 0.24), activities of daily living (MD = −1.51, 95% CI −4.91 to 1.89, *p* = 0.38), and motor complications (MD = −0.36, 95% CI −1.31 to 0.59, *p* = 0.46). mHealth intervention did not lead to a significant reduction in quality of life [standardized mean difference (SMD) = 0.04, 95% CI −0.20 to 0.28, *p* = 0.76], depression (SMD = −0.12, 95% CI −0.37 to 0.13, *p* = 0.34), cognition (MD = 0.37, 95% CI −0.34 to 1.09, *p* = 0.31), and balance (MD = 0.09, 95% CI −2.49 to 2.66, *p* = 0.95) (18).

For multiple sclerosis, most of the studies were focusing on cognitive function and fatigue. Concerning the efficacy, a small but significant effect was found for the use of mHealth for cognitive training [Standardized Mean Difference (SMD) = 0.28 (0.12; 0.45)] and a moderate effect for fatigue [SMD = 0.61 (0.47; 0.76)]. However, more replication studies are also needed as most of the mHealth have only been assessed in one single study (19).

### Countries in which the studies were performed

**Figure 1:**
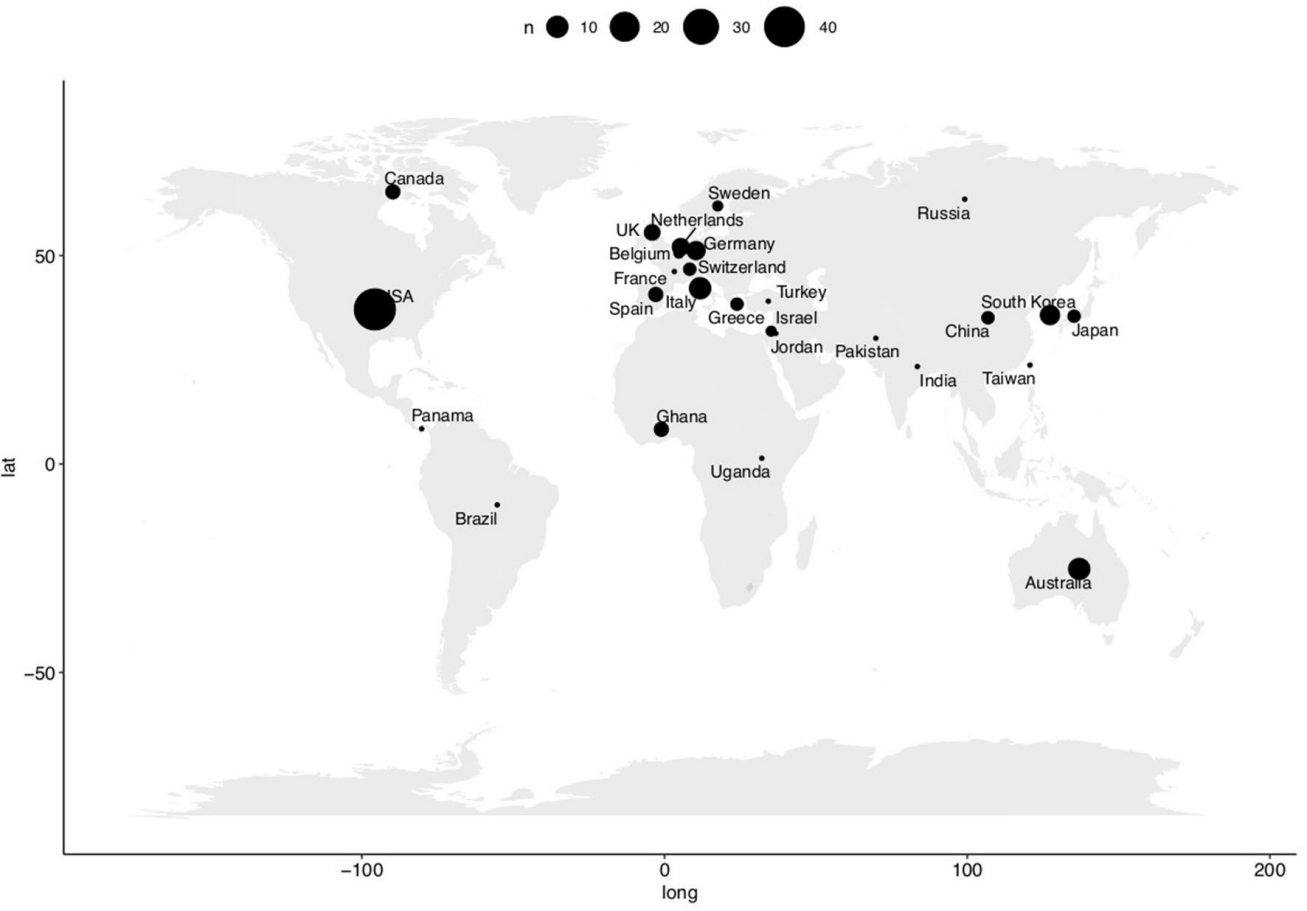
Breakdown of the included studies by country. The size of the bubbles is proportional to the number of studies performed in the countries.

We then extracted the number of studies and participants per country and plotted the results in Figure 1. The vast majority of the studies were done in the USA (32%), Europe (31%), and Asia (15%). Only six (4.5%) studies were performed in the BRICS (Brazil, Russia, India, China, and South Africa) and five (3.8%) studies in Africa (four in Ghana including three from the same team and one in Uganda). Of course, the results of this analysis must be seen in the light of one limitation: we performed an umbrella analysis, therefore, we are relying on the content of published systematic reviews (16–19) and may have missed the latest publications, small feasibility studies, or studies published in other languages than in English. However, the goal was to give a global vision of the current investigation of this technology, and we think that this method is well-adapted to draw up this assessment.

## Challenges

Incorporating mHealth solutions in the rehabilitation services in LMICs is a highly innovative and ambitious project; therefore, there are a lot of potential risks inherent to it. Here, we list the different risks and present strategies to mitigate these risks. The main challenges related to the development and use of new technology, including mHealth, can be analyzed at four different levels: the technology, the clinicians, the patients, and the policymakers.

From a public health perspective, different efforts must be done and synchronized to successfully integrate new interventions in the healthcare system, namely the macro, the meso, and the micro levels (24). The macro-level represents the legal, regulatory, and economic aspects; the meso-level concerns local health service and community; and the micro-level relates to the patients (25). Multilevel models that include relationships between proximal and distal determinants of health have substantially enhanced our knowledge of how health disparities occur (26). It is therefore important to incorporate these different dimensions in our analysis.

### Macro level

#### Policymakers

Robust governance structures are essential to ensure a cohesive and integrated approach to healthcare policy, planning, and delivery at all levels of the health system. Effective stewardship is required for governments to bear responsibility for safeguarding and increasing the welfare of their communities and building legitimacy and confidence among their constituents. Essential to good health governance is the stewardship role of the health ministry, which needs the identification and involvement of community stakeholders so that their views are heard and agreement is formed (27). It is also necessary to ensure that the diverse objectives of donor agencies and vertical programs addressing specific diseases do not impede the capacity of health systems to prioritize the health and wellbeing of the community as a whole. The long-term vision is that public healthcare policies must be taken at the political level, taking into account the financial and logistical realities of the field.

A significant challenge for the healthcare system is facilitating the discovery of safe and effective applications for healthcare practitioners and patients to create the most health benefit and guiding payer coverage choices when appropriate (28). Authors have suggested the notion of “prescriptable” mHealth applications, which are described as health apps that are presently accessible, have been shown to be successful, and are preferably stand-alone. When proved successful and accessible, standalone mHealth applications that do not need dedicated central servers and extra human resources may join other basic low-cost non-pharmaceutical therapies that general practitioners can prescribe (29).

### Meso level

#### Healthcare institutions

While it is very easy to share software for free, it is more difficult to do it with hardware or to provide a mobile connection to share the data between patients and clinicians and/or clinical centers. Concerning the problem of accessibility to the hardware (e.g., smartphones and tablets), the initiative from the “*OneBillion*” non-profit foundation should be highlighted here (30). They seek to deliver tablets preloaded with customized apps for teaching one billion youngsters to compute and read in their native language. This novel instructional method delivered individually through touch-screen tablets has been tested in a series of experiments conducted in Malawi, a low-income country in sub-Saharan Africa. The authors found that using this new interactive mHealth was efficient to support the learning of mathematics (Experiment 2) and reading (Experiment 3) (31). This highlights the fact that it is possible to deploy apps on a large scale even in low-income countries.

Despite this example, the main limitation is indeed probably the mobile connection, with 22% of Internet connectivity, Africa is the continent with the lowest level of coverage. Therefore, the new technology would be contextually adapted concerning local physical barriers (i.e., the accessibility of clinical centers) and taking into account the low level of internet coverage (i.e., promoting offline applications and a minimal number of transfers between the users and the clinicians).

The last but very important point is the sustainability of such type of intervention: the intervention must continue to be sustained without external resources (stakeholders) (32), and from a technological point of view, the apps developed and used must continue to work on older devices and operating systems. It is important to note here that the mHealth solutions should be used in combination with other treatments or at least under the supervision of one clinician (blended-care models) (33). Therefore, even though the first—and probably the most important—potential risk is indeed linked to the technological aspects: lack of power supply, Internet mobile coverage, technical troubleshooting capacity, and even if we are experiencing difficulties with the technology, the continuity of the care should be guaranteed by the healthcare professionals or the relatives and/or community (34).

#### Clinicians

From the professional's perspective, the most important point is probably education. First professionals need to be informed of the available mHealth apps and then should be perfectly aware of the possibilities offered by the technology, but also the limitations of these solutions. Given the limited amount of time available to patients, mHealth must be as straightforward as possible to avoid wasting consultation time and jeopardizing treatment. It is also of the utmost importance to raise awareness among local clinicians about the availability and importance of the validations of mHealth in the context of LMICs (13).

The gap between the number of scientific and clinical researchers in LMICs and their high burden of disease is exacerbated by the departure of up to 70% of scientists from their countries of birth for education and employment elsewhere (35). But as we have seen, all the mHealth have been validated in high-income countries (HICs) and studies need to be performed by clinicians and researchers to be sure that the results can be translated into LMICs. Reimbursement and implementation of this type of intervention on a large scale will only be possible if studies demonstrate its clinical utility in the field (36).

### Micro level

#### Patients and relatives

Most of the limitations may come from the technology's adoption by the patients. In LMICs, it represents a dual challenge: cultural and generational.

First, patients may have an important generation gap as older adults are not familiar with the use of new technology; furthermore, given the low education level and the high rate of illiteracy (in particular in rural areas), mHealth should not contain too much text. To improve adherence to diagnosis, therapy, and follow-up by SMS (37), beliefs, cultural features, and traditions should be included in the recommended solutions.

Furthermore, the established method should be unaffected by the subjects' educational level as much as feasible, there are also cultural and language impacts to consider. Attempting to solve the problem of aging using technology on a global scale is equally difficult. The usefulness of mHealth is influenced by four important areas of aging barriers: cognition, motivation, physical capacity, and perception (38).

## Call for action

A key challenge is moving mHealth from pilot projects to scalable national programs (39). The best approach to improve the use of mHealth in LMICs is to involve a highly multidisciplinary team to share the knowledge exchange. The project must not only focus on the clinics but should also include: technological development (e.g., development and use of portable technology that can be used without access to WIFI connection), building united networks (i.e., external power supply generator in the rehabilitation centers), and the education of healthcare professionals to share the knowledge.

At the WHO level, a rehabilitation competency framework has been developed to provide foundations for curricula for rehabilitation specialists (40). It is advocated to include mHealth tools related to rehabilitation in the competency framework. As a start of the discussion, a 10-point action plan proposed by the authors is presented in Table 1.

**Table 1:** Action plan to increase the use, validation, and implementation of mHealth solutions in LMICs: 10 key points with their potential threats and mitigation measures to overcome these limitations.

**Objectives**	**Description**	**Threats**	**Mitigation strategy**	**Target**			

				**Technology**	**Clinicians**	**Patients**	**Policymakers**
1. Availability	The development and implementation of mHealth (and more generally the eHealth) is strongly dependent on the coverage of stable Internet (mobile or fixed) connection and safe storage facilities (on-site or on the cloud)	•Lack of Internet coverage •Unstable connections •Lack of accessibility to the data •Safety issue (sensible data)	•Increasing Internet access points and base stations in urban and rural areas	**⊗**			**⊗**
2. Accessibility	Beside the internet connection, the hardware (i.e., smartphones or tablets) and the software should be available for both patients and clinicians	•Lack of access to the hardware •Incompatibility between available hardware (may be outdated) and the new version of the software (mHealth)	•Policymakers should help the digitalization of the society (hardware) •Developed should use basic library and support when developing the apps to allow them to be used on relatively old equipment	**⊗**			**⊗**
3. Adaptability	The solution should be adapted to take into consideration the cultural aspect as well as the perception and representation of the disease and its management	•The use of solutions developed in high income countries (i.e., Europe or USA) will lead to poor acceptance and use of these solutions	•Specific solutions must be developed involving local actors (clinicians and patients) or the existing solutions must be adapted •Cultural aspect, believes and representation of the diseases must be taken into consideration •mHealth must be available in local languages and dialects	**⊗**	**⊗**	**⊗**	
4. Validity	Research should be done on the field to determine the level of evidence supporting these interventions in the context of LMICs	•Considering that if the solutions have been validated in Europe and the USA, they are also validated in LMICs	•Local research to determine the level of acceptance (both by clinicians and patients) and the level of evidence of the mHealth solutions		**⊗**		**⊗**
5. Acceptability	The translation between research (point 4) and future daily clinical use must be ensured	•Solutions not adapted to the local context (Point 3) •Solutions not validated in the local context (Point 4)	•Development of culturally and socially accepted solution (Point 3)		**⊗**	**⊗**	
6. Affordability	The price of the proposed solutions, but also the running cost and maintenance costs must take into account the specific socio-demographic aspects	•Fee-based used of the mHealth. Paid solutions will only accentuate health inequalities instead of reducing them by making rehabilitation accessible to the greatest number	•Development of local or international initiatives to provide free services for the patients •Cost-efficiency study must be performed at the regional level				**⊗**
7. Usability	The applications should be user-friendly and bug-free in order to promote long-term use	•Applications too difficult for health professionals to use in daily clinical practice (both in terms of using the solutions and interpreting the data)	•Prototype must be developed with local actors (multiple iterations) •Feasibility studies to assess the level of acceptance (Point 5) and ease of use before testing the programs on a large scale	**⊗**			
8. Education	Healthcare education and training is one of the keys to the success of the implementation of new solutions in rehabilitation, and in the healthcare sector in general. The professionals should be perfectly aware of the limitations and potential of the new applications	•Healthcare professionals may have too high expectations if they do not know the limits of the mHealth •They may not use the apps to their full potential if they are not aware of all the services offered	•Healthcare professionals must be well-trained to provide the most convincing and clear explanations possible to patients to encourage them to use these solutions •Specific educational program must be developed to train healthcare professionals		**⊗**		**⊗**WHO^*^
9. Literacy	Education is also the key for the patient. Information should be provided to the patients about the importance of using mHealth to improve rehabilitation outcomes	•Patients are not sufficiently aware of the importance of regular follow-up during the rehabilitation •Patients do not see the value of regular revalidation exercises between sessions supervised by the rehabilitation specialist	•Well-trained professionals who give clear explanations on how to use the applications and the importance of these applications for the rehabilitation process (Point 8) •Feedback must be provide in the mHealth to show the evolution of the patients to stimulate them to keep using the apps •Education module should be available in the mHealth to inform the patients about their conditions			**⊗**	**⊗**WHO^*^
10. Sustainability	The proposed solution should be implemented on a long-term basis. The acceptability of a new technology is a long-term process and once the patients start using the solution, they should be assured of continuing to use it for the duration of their rehabilitation program or follow-up in the case of chronic pathologies.	•Short term research project •Low interest from the companies	•The implementation of mHealth (and more broadly eHealth) must be the result of a long-term policy and vision •The development of stable and sustainable infrastructure (Point 1) •Free distribution of the software for patients (Point 6) and with limited fees for healthcare professionals and clinical centers		**⊗**		**⊗**

* WHO competency framework (31).

From the technological perspective, there is an urgent need to develop strong local infrastructures to help the implementation of mHealth in daily practice. Such development should be done using the North-South consortium to develop inter and multidisciplinary collaborations. Such regional centers (local hubs) are important not only to provide care to the patients but also to facilitate education and will also enable the development of research programs (41), which are essential to test and validate the use of mHealth in the LMICs and define the level of evidence associated with these interventions.

For healthcare professionals, training is also important to get familiar with new technology (hardware and software), therapeutic applications, and associated dangers; familiarity with instruments for identifying patients who might benefit from mHealth interventions (e.g., every patient could respond differently to them, and some patients could be at a higher risk of non-compliance). An important aspect of education and training should be to promote research. It is indeed of the utmost importance to develop local scientific research capacity and generate evidence supporting the use of the new solutions taking into account the local and cultural specificities but also the technical constraints (e.g., infrastructure and low-quality mobile connection). Currently, studies describing newly developed mHealth and demonstrating the effectiveness of the technological-supported interventions are performed in high-income countries. Therefore, the translation of these results to the context of LMICs is not straightforward, and there is an urgent need to develop local scientific evidence. It is urgent to determine first the feasibility and acceptability (of adapted technology) of both the patients and the clinicians and then to determine the local level of evidence at a regional level (particularly important in the context of evidence-based practice), as other rehabilitation targets may apply to LMICs compared to HIC (42).

Improving both clinical management and research can be done through the implementation of a clinical research mentorship program (43). The development of these new mHealth technologies and solutions, allowing for continuous patient monitoring and follow-up (44, 45), is also a unique opportunity to develop more individualized rehabilitation (46–48). According to the WHO, people-centered care is a much broader concept than only actively involving the patient in the care. It is defined by WHO as “an approach to care that consciously adopts individuals,” carers', families', and communities' perspectives as participants in, and beneficiaries of, trusted health systems that are organized around the comprehensive needs of people rather than individual diseases, and respects social preferences' (4). People-centered care also necessitates that patients have the knowledge and support they require to make decisions and engage in their care and that caregivers perform optimally within a supportive working environment.

From the patient's perspective, to promote adherence to rehabilitation and assessment, mHealth beliefs, cultural features, and traditions should be included in the apps (26). In addition, there are cultural and linguistic impacts to consider when developing or adapting these apps (49). Integrating a cultural component into mHealth boosts user engagement and participation in the training component (50).

At the macro level, the WHO has developed a tool for systematic assessment of the rehabilitation situation at the country level (51). It is advocated also that mHealth solutions are being incorporated in the assessment and most importantly subsequent future planning of goals in further developing rehabilitation services.

It is also of the utmost importance to fully integrate the new solutions in the care to be able to propose, as highlighted by the WHO, an integrated healthcare service. Integrated health services are defined by the WHO as “health services that are managed and delivered so that people receive a continuum of health promotion, disease prevention, diagnosis, treatment, disease-management, rehabilitation and palliative care services, coordinated across the different levels and sites of care within and beyond the health sector, and according to their needs throughout the life course” (4). A schematic representation of the different levels of governance (macro, meso, and micro) and the alignment with the strategy of the WHO to develop a more people-centered vision of care is presented in Supplementary Figure 1.

## Conclusion

New technology must be used to enhance global health and mitigate the burden of chronic diseases and the lack of rehabilitation professionals. While new technologies cannot address all of the LMIC's health concerns, in the absence of effective and accessible therapies for rehabilitation, these mHealth applications may be very valuable. Researchers, rehabilitation staff, healthcare workers, physicians, and app developers will need to cooperate to produce creative, effective solutions tailored to rehabilitation taking into account both cultural and infrastructural aspects of LMICs. While technological solutions are available for the most important disorders, they are currently not being used in LMIC, especially in Africa, as shown in this work.

## Data availability statement

The original contributions presented in the study are included in the article/Supplementary material, further inquiries can be directed to the corresponding author.

## Author contributions

BB wrote the first version of this paper. All authors contributed to the article and approved the submitted version.

## Supplementary material

Supplementary Figure 1 Potential response shift implications for use of mHealth data at micro-, meso-, and macro-levels of healthcare decision-making.

Supplementary Table 1 Thresholds used to define the different economies according to the World Bank Atlas method and countries.
