# Supplementary figures and images for: Mobile health solutions: An opportunity for rehabilitation in low- and middle income countries?
Source: Front Public Health. 2023 Jan 24;10:1072322. doi: 10.3389/fpubh.2022.1072322 (PMC9902940; doi:10.3389/fpubh.2022.1072322)

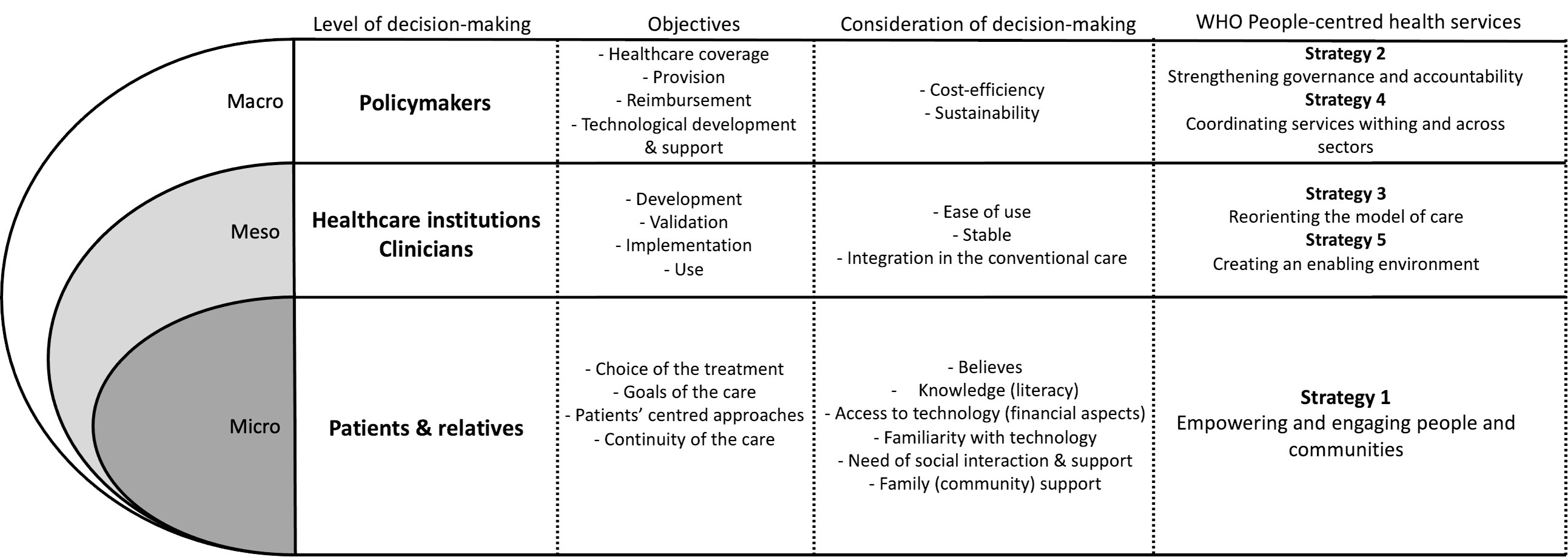

Supplement: Supplementary Figure 1 — Potential response shift implications for use of mHealth data at micro-, meso-, and macro-levels of healthcare decision-making. [file Image_1.JPEG]
