# Supplementary material for: Mobile health solutions: An opportunity for rehabilitation in low- and middle income countries?
Source: Front Public Health. 2023 Jan 24;10:1072322. doi: 10.3389/fpubh.2022.1072322 (PMC9902940; doi:10.3389/fpubh.2022.1072322)
Supplement: Supplementary Table 1 — Thresholds used to define the different economies according to the World Bank Atlas method and countries. [file Table_1.docx]

**Supplementary Table 1**: Thresholds used to define the different economies according to the World Bank Atlas method and countries

| **Category** | **Definition** | **Countries** |
| --- | --- | --- |
| Low-income country | Low-income economies are defined as those with a GNI per capita, calculated using the [World Bank Atlas method](https://datahelpdesk.worldbank.org/knowledgebase/articles/378832-what-is-the-world-bank-atlas-method), of $1,045 or less in 2020 | Afghanistan, Burkina Faso, Burundi, Central African Republic, Chad, Eritrea, Ethiopia, Gambia, Guinea, Guinea-Bissau, Democratic People’s Republic of Korea, Liberia, Madagascar, Malawi, Mali, Mozambique, Niger, Rwanda, Sierra Leone, Somalia, South Sudan, Sudan, Syrian Arab Republic, Togo, Uganda, Yemen |
| Lower middle-income country | Lower middle-income economies are those with a GNI per capita between $1,046 and $4,095. | Algeria, Angola, Benin, Bangladesh, Belize, Bolivia, Bhutan, Cambodia, Cameroon, Côte d'Ivoire, Comoros, Cabo Verde, Djibouti, Egypt; Micronesia, Ghana, Honduras, Haiti, Indonesia, India, Islamic Republic of Iran, Kenya, Kyrgyz Republic, , Kiribati, Lao PDR, Sri Lanka, Lesotho, Morocco, Myanmar, Mongolia, Mauritania, Nigeria, Nicaragua, Nepal, Pakistan, Philippines, Papua New Guinea, West Bank and Gaza, Senegal, Solomon Islands, El Salvador, São Tomé and Principe, Eswatini, Tajikistan, Timor-Leste, Tunisia, Tanzania, Ukraine, Uzbekistan, Vietnam, Vanuatu, Samoa, Zambia, Zimbabwe |
| Upper middle-income country | Upper middle-income economies are those with a GNI per capita between $4,096 and $12,695 | Albania, American Samoa, Argentina, Armenia, Azerbaijan, Belarus, Bosnia and Herzegovina, Botswana, Brazil, Bulgaria, China, Colombia, Costa-Rico, Cuba, Dominica, Dominican Republic, Equatorial Guinea, Ecuador, Fiji, Gabon, Georgia, Grenada, Guatemala, Guyana, Iraq, Jamaica, Jordan, Kazakhstan, Kosovo, Lebanon, Libya, Malaysia, Maldives, Mashal Islands, Mauritius, Mexico, Moldova, Montenegro, Namibia, North Macedonia, Panama, Paraguay, Peru, Romania, Russian Federation, Serbia, South Africa, St Lucia, St. Vincent and the Grenadines, Suriname, Thailand, Tonga, Turkey, Turkmenistan, Tuvalu |
| High-income country | High-income economies are those with a GNI per capita of $12,696 or more | Andorra, Antigua and Barbuda, Aruba, Australia, Austria, Bahamas, Bahrain, Barbados, Belgium, Bermuda, British Virgin islands, Brunei Darussalam, Canada, Cayman Islands, Channel Islands, Chile, Croatia, Curaçao, Cyprus, Czech Republic, Denmark, Estonia, Faroe Islands, Finland, France, French Polynesia, Germany, Gibraltar, Greece, Greenland, Guam, Hong Kong SAR (China), Hungary, Iceland, Ireland, Isle of Man, Israel, Italy, Japan, Korea (Rep), Kuwait, Latvia, Liechtenstein, Lithuania, Luxembourg, Macao SAR (China), Malta, Moncaco, Nauru, Netherlands, New Caledonia, New Zeeland, Northern Mariana Islands, Norway, Oman, Palau, Poland, Portugal, Puerto Rico, Qatar, San Marino, Saudi Arabia, Seychelles, Singapore, Sint Maarten (Dutch part), Slovak Republic, Slovenia, Spain, St. Kitts and Nevis, St. Martin (French part), Sweden, Switzerland, Taiwan (China), Trinidad and Tobago, Turks and Caicos Islands, United Arab Emirates, United Kingdom, Unites States, Uruguay, Virgin Islands (USA) |

1. World Bank. World Bank Country and Lending Groups. (2021). https://datahelpdesk.worldbank.org/knowledgebase/articles/906519-world-bank-country-and-lending-groups
